# Evaluating the Safety of Imidacloprid FS Seed Treatment Use in Potato Production: A Case Study from China

**DOI:** 10.3390/molecules29143362

**Published:** 2024-07-17

**Authors:** Abdul Kaium, Chi Wu, Yanli Man, Xingang Liu, Fengshou Dong, Youngquan Zheng

**Affiliations:** 1 State Key Laboratory for Biology of Plant Disease and Insect Pests, Institute of Plant Protection, Chinese Academy of Agricultural Sciences, No. 2, West Yuan-Ming-Yuan Road, Beijing 100193, China; kaium.agrichemistry@sau.edu.bd (A.K.); wuchi@caas.cn (C.W.); sdstzsmyl@163.com (Y.M.); fsdong@ippcaas.cn (F.D.); yqzheng@ippcaas.cn (Y.Z.); 2 Department of Agricultural Chemistry, Sher-e-Bangla Agricultural University, Dhaka 1207, Bangladesh

**Keywords:** imidacloprid FS, potato, soil, residue behavior, dietary risk, ecological risk

## Abstract

This study evaluated the residue behavior and dissipation dynamics of a new imidacloprid FS 600 seed treatment in potato cultivation systems in Shandong and Jilin, China. Sensitive and accurate UPLC-MS/MS methods were established to quantify imidacloprid residues in potatoes, potato plants, and soil. Results showed that imidacloprid dissipation followed a first-order kinetic model, with half-lives ranging from 6.9 to 26.7 days in plants and 19.8 to 28.9 days in soil. At harvest, the highest average residues in potatoes and soil were 0.778 mg/kg and 0.149 mg/kg, respectively. The dietary risk assessment indicated a chronic risk quotient (CRQ) of 39.73% for adults, indicating minimal risk to human consumers, while the ecological risk quotient (ERQ) and ecotoxicity exposure ratio (TER) revealed low to moderate toxicity to earthworms, warranting caution in the use of this formulation. This research provides valuable data for assessing the safety of imidacloprid FS seed treatment in potato cultivation.

## 1. Introduction

Modern agriculture has made extensive use of imidacloprid, a systemic neonicotinoid insecticide, to protect crops from pest infestations. It is a game-changer as an insecticide due to its low toxicity to mammals, excellent effectiveness, and versatility in use [1,2]. In the last 30 years, its non-repellent properties and high effectiveness have made it increasingly popular [3]. Despite its agricultural benefits, the extensive use of imidacloprid has raised concerns about its environmental impact, particularly on non-target organisms such as pollinators, aquatic life, and soil microorganisms [4,5]. For instance, it has been shown to affect pollinators such as bees, leading to reduced foraging efficiency, impaired navigation, and colony collapse [4]. These concerns have led to restrictions on the use of imidacloprid insecticides in some regions, highlighting the need for comprehensive risk assessments to evaluate the safety of their use in specific agricultural contexts [6]. Moreover, its relatively high water solubility (0.61 g/L at 20 °C) raises concerns about its potential to leach into groundwater, posing risks to aquatic ecosystems and drinking water sources [7]. Therefore, careful dosage management is crucial to prevent the subsurface from becoming a sink for this pesticide, ensuring both agricultural productivity and environmental protection. Therefore, recent years have seen a surge in its use as a direct seed treatment, showing promising results in controlling pests like jassids, thrips, and whiteflies in potatoes [8,9]. Moreover, its application as a seed treatment formulation (FS) has gained popularity due to its ability to protect crops against soil-borne pests during critical early growth stages [10].

The agricultural sector in China plays a critical role in ensuring food security and supporting the livelihoods of millions of farmers. Potatoes (Solanum tuberosum) are a significant crop, with China being the world’s leading producer, accounting for over 25% of global production [11,12,13]. Despite its prominence, the production of potatoes in China lags behind the global average, primarily hindered by various diseases and insect pests [14,15]. In addressing these challenges, imidacloprid has been used extensively in various crops, including potatoes, to manage pests effectively [15,16]. This concern is heightened in China, where extensive agricultural activities involve the use of this pesticide. Various studies have explored the implications of imidacloprid use, including its uptake in plants, persistence in soil, and potential risks to non-target soil organisms [17,18]. The dissipation behavior of imidacloprid in agricultural soils and its subsequent residues in crops are critical parameters that influence its environmental impact and food safety [10]. Previous studies have shown that the dissipation rate of imidacloprid in the soil can be influenced by several factors, including soil type, temperature, moisture, and microbial activity [19,20]. Understanding the degradation kinetics and residue levels of imidacloprid in potato fields is essential for assessing its long-term environmental impact and ensuring that its use complies with safety regulations.

The risk assessment of pesticide residues is a key component of sustainable agricultural practices. It involves evaluating the potential adverse effects on human health and the environment, considering both acute and chronic exposure scenarios [6]. Recent studies have shown that exposure to imidacloprid in agricultural settings has been associated with measurable concentrations of this pesticide in human urine, suggesting a potential health risk for those close to treated fields [18]. Despite the critical role of the new FS seed treatment formulation of imidacloprid in agriculture, there is a noticeable gap in comprehensive studies focused on its dissipation and residue behavior, specifically in China’s potato fields. Therefore, a comprehensive assessment of the risks associated with imidacloprid use in potato fields is imperative to develop guidelines that minimize its negative impacts while maintaining its pest control benefits. This case study from China aims to provide a comprehensive evaluation of imidacloprid FS seed treatment in potato production, focusing on residue dynamics, environmental impact, and safety for human exposure. The findings will contribute to the development of safer agricultural practices and regulatory policies for pesticide use in China.

## 2. Results and Discussion

### 2.1. Quality Control of the Analytical Method

#### 2.1.1. Specificity

**Figure 1:**
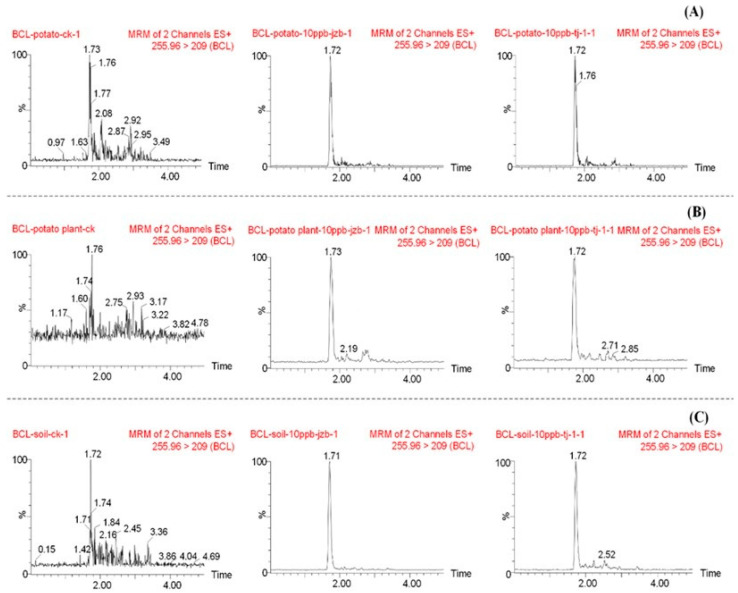
Representative UPLC-MS/MS chromatograms of imidacloprid. (**A**) Potato. (**B**) Potato plant. (**C**) Soil. Each panel displays chromatograms for a blank matrix sample, a matrix-matched standard solution, and a spiked sample (0.01 mg/kg).

The instrumental method’s specificity was assessed using blank and spiked samples (potato, potato plant, and soil). Imidacloprid consistently eluted at a retention time of approximately 1.7 min under the established chromatographic conditions (Figure 1). This unique retention time for the analyte demonstrates the method’s ability to effectively separate and identify imidacloprid in diverse matrices.

#### 2.1.2. Linearity

Linearity was assessed using seven imidacloprid standard solutions (0.005–2.0 mg/kg), prepared via serial dilution in acetonitrile or matrix extracts. Strong linear responses were observed for all matrices, with correlation coefficients (R^2^) consistently exceeding 0.9950 (Table 1). This exceptional linearity surpasses the European Commission’s guidelines, underscoring the method’s robust reliability for the accurate quantification of imidacloprid residues.

#### 2.1.3. Sensitivity

Matrix-matched recovery experiments established the limit of quantification (LOQ) for imidacloprid in potatoes and soil at 0.01 mg/kg, aligning with the European Commission’s latest maximum residue limit (MRL) [21] and considerably lower than China’s MRL of 0.5 mg/kg for potatoes (Table 1). The limit of detection (LOD) was determined to be 0.005 mg/kg, highlighting the method’s sensitivity. Both LOD and LOQ values complied with the SANTE 2021 method validation guidelines, further validating the method’s robustness and suitability for residue analysis [22].

#### 2.1.4. Matrix Effect (ME)

Matrix effects (MEs), a common phenomenon in chromatographic analysis with mass spectrometry, can significantly influence analyte quantification [23]. Following SANTE guidelines (2021), MEs were categorized as soft (≤20%), medium (20–50%), or strong (>50%) [22,24]. Our study revealed strong MEs (>50%) for potato, potato plant, and soil samples, indicating significant ion suppression in the QuEChERS-based UPLC-MS/MS method. To address this, matrix-matched calibration curves were employed, ensuring the accurate quantification of imidacloprid residues. This approach is essential in mitigating ion suppression, a prevalent issue in LC-MS/MS analysis due to factors like the electrospray ionization source, sample type, preparation process, and mobile phase composition [23,24].

#### 2.1.5. Accuracy and Precision

Recovery experiments were conducted at three spiking levels (0.01, 0.1, and 0.5 mg/kg) for imidacloprid in potato, potato plant, and soil samples. Mean recovery rates ranged from 74.32% to 89.00% in potatoes, 70.78% to 79.26% in potato plants, and 84.50% to 95.62% in soil (Table 2). Relative standard deviations (RSDs), reflecting method precision, ranged from 1.97% to 17.41%. These results demonstrate the method’s accuracy and reproducibility across diverse matrices, aligning with the European Commission’s method validation criteria [22]

### 2.2. Dissipation Studies

#### 2.2.1. Dissipation Dynamics of Imidacloprid in Potato Plants

**Figure 2:**
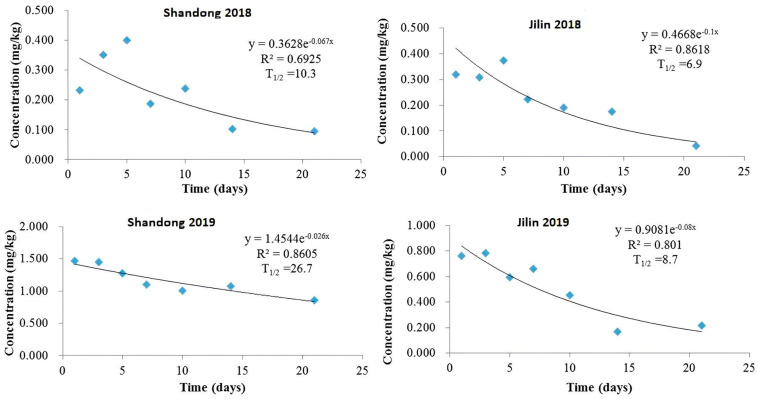
Degradation dynamics of imidacloprid in potato plants in Shandong and Jilin province experimental sites in 2018 and 2019.

Our study demonstrated that imidacloprid dissipation in potato plants followed a first-order kinetic model. In 2018, using 1.5 times the recommended dosage, average residues in Shandong and Jilin declined from 0.233 and 0.320 mg/kg on day 1 to 0.095 and 0.041 mg/kg by day 21, respectively. This corresponded to half-lives of 10.3 days (R^2^ = 0.6925) and 6.9 days (R^2^ = 0.8618) (Figure 2). The higher dosage applied in 2019 led to initial residues of 1.468 and 0.763 mg/kg, decreasing to 0.861 and 0.214 mg/kg by day 21, with extended half-lives of 26.7 days (R^2^ = 0.8605) and 8.7 days (R^2^ = 0.801) for Shandong and Jilin, respectively (Figure 2). This indicates slower dissipation in 2019 compared to 2018.

The observed variability in half-lives and dissipation rates likely stems from differences in soil physiochemical properties (pH, organic matter content), climatic conditions (temperature, rainfall, humidity, sunshine hours, UV irradiation), and plant surface characteristics, as previously reported [23,25]. These factors influence pesticide solubility, translocation, and degradation kinetics within plants.

#### 2.2.2. Dissipation Dynamics of Imidacloprid in Soil

**Figure 3:**
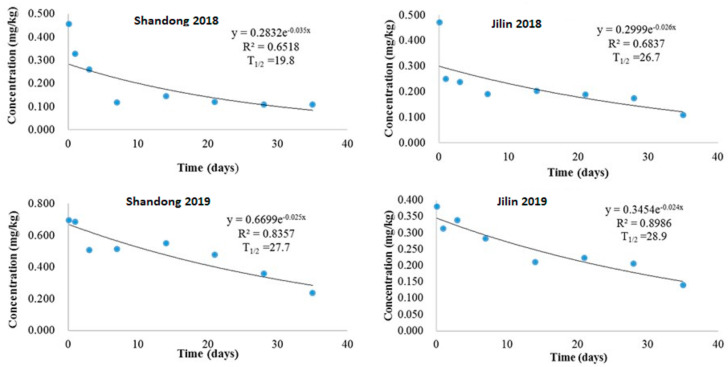
Imidacloprid degradation dynamics in potato field soil of Shandong and Jilin provinces in 2018 and 2019.

Our study demonstrated that the dissipation of imidacloprid in potato field soil from Shandong and Jilin provinces followed an exponential decay pattern. In 2018, after a 1.5× recommended dosage application, initial concentrations in Shandong (0.456 mg/kg) and Jilin (0.472 mg/kg) declined to 0.108 mg/kg and 0.109 mg/kg, respectively, over 35 days. This resulted in half-lives of 19.8 days (Shandong) and 26.7 days (Jilin), with dissipation equations of y = 0.2832e^−0.035x^ (R^2^ = 0.6518) and y = 0.2998e^−0.026x^ (R^2^ = 0.6837), respectively (Figure 3). Similarly, in 2019, initial concentrations of 0.696 mg/kg (Shandong) and 0.381 mg/kg (Jilin) decreased to 0.237 mg/kg and 0.140 mg/kg, respectively, after 35 days. Half-lives were slightly longer at 27.7 days (Shandong) and 28.9 days (Jilin), with the dissipation equations being y = 0.6699e^−0.025x^ (R^2^ = 0.8357) and y = 0.3454e^−0.024x^ (R^2^ = 0.9876), respectively (Figure 3).

The observed variability in imidacloprid half-life and dissipation rates is likely due to diverse soil types and a complex interplay of physicochemical and climatic factors. These include temperature, rainfall, humidity, sunshine hours, pH, organic matter content, and UV irradiation [26,27]. Recent research corroborates these findings, demonstrating the influence of application dose on imidacloprid half-lives in rice soil [28] and the impact of soil salinity on dissipation dynamics [29]. Furthermore, consistent with previous studies, imidacloprid dissipation is notably faster in plant tissues compared to soil. This discrepancy can be attributed to the distinct mechanisms of dissipation and degradation operating in each matrix [20,30].

### 2.3. Terminal Residue Studies

#### 2.3.1. Terminal Residue in Potato

Final imidacloprid residues in potatoes varied depending on dosage and year (Table 3). In 2018, residues in Shandong and Jilin provinces were 0.059 and 0.029 mg/kg, respectively, at the recommended dosage (50 mL/100 kg seed tuber), increasing to 0.077 and 0.033 mg/kg at the higher dosage (75 mL/100 kg). Notably, residues increased significantly in 2019, reaching 0.238 and 0.104 mg/kg in Shandong and Jilin, respectively, at the recommended dosage, and 0.778 and 0.075 mg/kg at the higher dosage.

The observed variations in imidacloprid residue levels align with previous findings. Trapp (2000) highlighted the influence of a pesticide’s water solubility on its translocation within plants, suggesting a potential explanation for the differential accumulation of potato components [25]. Similar dynamics were observed by Yu et al. (2019) in other crops, where imidacloprid residues followed first-order kinetics and posed minimal health risks under standard usage [16]. This aligns with our results, including the increase in residue levels noted in 2019. Additionally, the inter-annual variability in residues could be attributed to factors such as differing environmental conditions and potato varieties, as emphasized by Bekolo et al. (2015) regarding imidacloprid in potato crops [31].

#### 2.3.2. Terminal Residue in Field Soil

This study examined the final residues of imidacloprid in potatoes after applying different dosages as a seed dressing. The results, as shown in Table 4, indicate that in 2018, the residues in potatoes from Shandong and Jilin provinces were 0.059 mg/kg and 0.029 mg/kg, respectively, at the recommended dosage of 50 mL of imidacloprid per 100 kg of seed tuber. For the higher-dosage trial (75 mL/100 kg seed tuber), the residues were 0.077 mg/kg and 0.033 mg/kg, respectively. In 2019, there was a notable increase in residues, with 0.238 mg/kg and 0.104 mg/kg in Shandong and Jilin, respectively, at the recommended dosage, and significantly higher residues of 0.778 mg/kg and 0.075 mg/kg at the higher dosage.

The variation in imidacloprid residues observed between the two provinces underscores the influence of soil properties, climate, and agricultural practices on the soil’s environmental fate, as previously reported [4,5]. The lower residue levels in Jilin soils compared to Shandong could be attributed to factors such as soil type, moisture content, and microbial activity, which are known to affect pesticide degradation rates [20,32]. Additionally, formulation type may also influence residue dynamics, as evidenced by the faster degradation of nano-imidacloprid formulations compared to traditional ones [33]. Moreover, environmental factors like sunlight exposure have been shown to significantly impact imidacloprid persistence, with UV radiation accelerating its degradation in soil [34]. These findings highlight the complex interplay of factors that govern imidacloprid’s environmental fate and emphasize the importance of considering regional variations in risk assessment and management strategies.

### 2.4. Dietary Risk Assessment

Dietary risk assessment, employing a risk quotient (RQ) approach, revealed minimal risk associated with imidacloprid residues from FS seed treatment formulation in potatoes. The calculated RQ value of 39.73% (Table 5), derived from the supervised field trial median residue (STMR) of 0.239 mg/kg and theoretical maximum daily intake (TMDI) of 1.5016 mg, is low and can be considered acceptable within regulatory limits. This indicates that dietary exposure to imidacloprid through potato consumption is unlikely to pose a significant health risk.

These findings align with previous research. Cang et al. (2018) also reported RQ values below 1 for imidacloprid on strawberries, suggesting consistent safety across different crops [35]. Similarly, Yu et al. (2019) found terminal imidacloprid residues in Zizania latifolia and purple sweet potato to be well below risk thresholds [16]. These studies collectively reinforce the notion that imidacloprid FS seed treatment formulation, when used as recommended, poses minimal risk to human health. The present study’s RQ value of less than 40% adheres to both international and Chinese food safety standards, further affirming the low dietary risk associated with imidacloprid residues in potatoes. This is consistent with the work of Benbrook and Davis (2020), emphasizing the importance of rigorous residue analysis in ensuring food safety [36].

### 2.5. Ecological Risk Assessment

#### 2.5.1. Ecological Risk Quotient (RQ)

**Figure 4:**
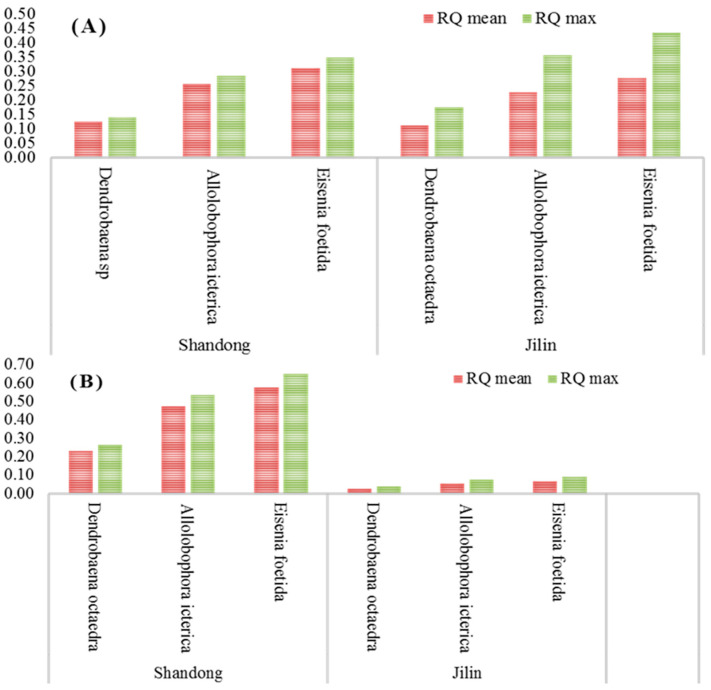
Ecological risk quotient (RQ) of imidacloprid FS at the recommended dose (**A**) and higher dose (**B**) applied to potato field soil in Shandong and Jilin provinces, China.

**Figure 5:**
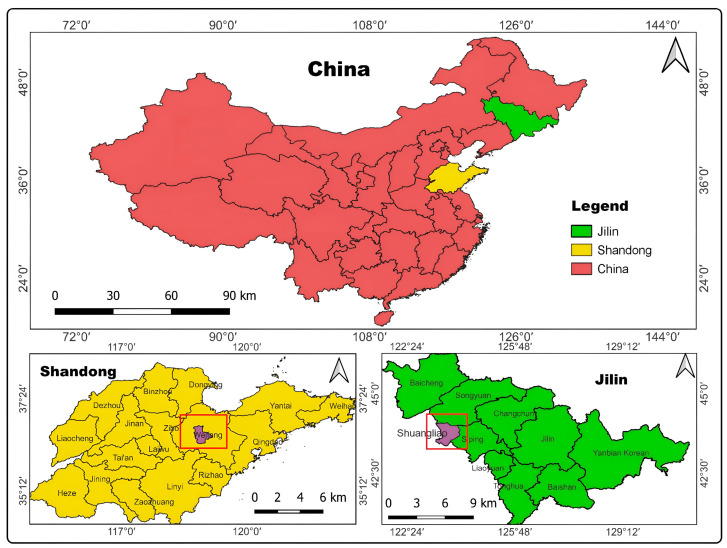
Study sites for imidacloprid FS in Chinese potato cultivation systems.

This study focused on assessing the ecological impact of imidacloprid FS seed treatment formulation on earthworms, key bioindicators of soil health, in potato fields across Shandong and Jilin provinces in China. Ecological risk quotients (RQ) were calculated based on imidacloprid residue levels in soil to estimate the potential risk to various earthworm species. For imidacloprid FS seed treatment used at recommended dosages, the RQmean and RQmax across various earthworm species ranged from 0.11 to 0.43 in both provinces (Figure 4). In scenarios involving higher dosages, these values spanned between 0.03 and 0.65 (Figure 5). Notably, both mean and maximum RQ values significantly exceeded 0.1 but remained below 1.0, except in cases of higher imidacloprid dosages in Jilin province. These results suggest that imidacloprid FS seed treatment in these potato-growing regions poses a low to moderate ecological risk to earthworms.

This aligns with previous studies that have reported similar RQ values for imidacloprid in other crops and regions [37]. However, it is essential to acknowledge that the ecological risk of imidacloprid can vary depending on various factors, including soil properties, application rates, and the sensitivity of different earthworm species [38]. Implementing integrated pest management strategies, such as crop rotation and biological control, can help minimize the reliance on chemical insecticides and mitigate potential ecological impacts [39].

#### 2.5.2. Ecological Toxicity Exposure Ratios (TER)

The Toxicity Exposure Ratio (TER) provides further insights into the ecological safety of imidacloprid FS use. The European Commission (EC) sets trigger points of 5 and 10 for chronic and acute toxicity data, respectively [40]. In our study, TERmean and TERmax values ranged from 1.53 to 8.91, suggesting potential acute to chronic risk to earthworm species in both standard and higher dose experiments in Shandong and Jilin. Notably, higher dose experiments in Jilin province exhibited TER values (10.95 to 36.77) significantly exceeding the trigger point, suggesting negligible risk to earthworm species due to the faster degradation of imidacloprid facilitated by weather and soil characteristics (Table 6). These results revealed an acute to chronic risk continuum for different earthworm species under standard imidacloprid usage in these regions.

Previous studies have reported that the toxicity of imidacloprid to earthworms varies across different soil types. In tropical soils, imidacloprid exhibits higher toxicity, especially in sandy soils compared to clayey soils, underscoring the need to consider soil type in ecological risk assessments [41]. Furthermore, different species of earthworms exhibit varying levels of sensitivity to imidacloprid. Studies have shown that certain earthworm species are more susceptible to the toxic effects of imidacloprid than others, highlighting the importance of species-specific risk assessments [42].

## 3. Materials and Methods

### 3.1. Reagents and Materials

Imidacloprid standard (98.8% purity) was provided by Bayer CropScience (Leverkusen, Germany). LC-grade methanol and acetonitrile for sample preparation and mobile phase solvents were purchased from Sigma-Aldrich, Darmstadt, Germany. Dispersive solid-phase extraction reagents, including sodium chloride (NaCl) and anhydrous magnesium sulphate (MgSO_4_), as well as analytical-grade reagents like potassium permanganate, sodium bisulfite, sodium hydroxide, ethyl acetate, concentrated sulfuric acid, and formic acid, were purchased from Beijing Chemical Company, Beijing, China. Dispersive solid-phase cleanup sorbents, including primary secondary amine (PSA), graphitized carbon black (GCB), octadecyl (C18), and Florisil, were purchased from Bonna-Agela Technologies, Tianjin, China. Additionally, 50 mL polypropylene centrifuge tubes, 2 mL Eppendorf tubes, 0.22 μm polytetrafluoroethylene (PTFE) filters, and 2 mL vials for LC analysis were also supplied by Bonna-Agela Technologies, Tianjin, China.

### 3.2. Preparation of Standard Solution

Imidacloprid standards were accurately weighed and dissolved in acetonitrile to prepare a 100 mg/L stock solution. This stock solution was then serially diluted with acetonitrile to produce standard working solutions at concentrations of 2 mg/L, 1 mg/L, 0.5 mg/L, 0.1 mg/L, 0.05 mg/L, 0.01 mg/L, and 0.005 mg/L.

### 3.3. Experimental Sites and Times

Field trials were conducted in 2018–2019 at experimental sites in Weifang (Shandong Province) and Shuangliao (Jilin Province) to assess final imidacloprid residues and understand their environmental fate and residual behavior following FS seed treatment formulation (Figure 5). Both virus-free and Holland No. 7 potato cultivars were grown at these experimental sites. Detailed summaries of these field experiments are provided in Supplementary Table S1.

### 3.4. Soil Type and Climatic Conditions

The experimental sites in Shandong Province, characterized by a semi-humid monsoon climate, featured fluvo-aquic soil with a pH of 6.9 and an organic matter content of 2.0%. This region experiences four distinct seasons, including cold winters, hot summers, and significant rainfall, with annual sunshine hours ranging from 2300 to 2700 and average temperatures at around 21 °C during the experiments. In contrast, Jilin Province, under a temperate continental monsoon climate, presented black soil with a pH of 6.4 and 2.3% organic matter. This area also experiences four distinct seasons, with hot, rainy summers and cold, dry winters. Yearly sunshine hours vary from 2259 to 3016, and the average temperature during the experiments was around 23 °C. For both sites, detailed climate and soil characteristics are provided in Supplementary Table S2.

### 3.5. Design of the Field Experiment

Field experiments were conducted across two locations in China, Shandong and Jilin provinces, using 30 m^2^ plots. Each location comprised three replicated treatment plots and a control plot, each separated by protective buffer zones. Imidacloprid FS, a seed treatment formulation at a concentration of 600 g/L, was used for grub control. Two dosage levels were investigated: a low dose of 50 mL FS per 100 kg seed potato (30 g active ingredient) and a high dose of 75 mL formulation per 100 kg seed potato (45 g active ingredient, 1.5 times the recommended dose). For the dissipation dynamics study, the high dose was applied once to both the potato and the soil. Soil experiments included a control field treated with the high-dose imidacloprid FS mixed with soil but without potato planting. Additionally, six plots received either a low or high dose for residue analysis. The imidacloprid FS was diluted with water to a final volume of 5–10 mL per kilogram of seed tuber and applied once. Detailed information regarding dosages and sample collection can be found in Supplementary Tables S1 and S3.

### 3.6. Sample Collection

To investigate imidacloprid dissipation, potato plant and soil samples were collected randomly from treated plots according to the “Pesticide Residue Test Guidelines” (NY/T 788-2004). Potato plant samples, each weighing at least 2 kg, were collected on days 1, 3, 5, 7, 10, 14, and 21 post-applications. A 300 g subsample from each was retained and stored at −20 °C. Soil samples (0–10 cm in depth), approximately 1–2 kg each, were collected using a soil drill before spraying, 2 h after spraying, and on days 1, 3, 7, 14, 21, 28, and 35 post-applications. Each soil sample was cleaned of debris and reduced to 300 g for analysis. At harvest, for the final residue analysis, whole potato and soil samples (0–15 cm in depth), weighing at least 1 kg each, were collected. Each treatment was replicated three times and included a blank control. All field samples were transported to the laboratory within 8 h of collection. Potato and plant samples were immediately chopped, homogenized, and frozen at −20 °C.

### 3.7. Sample Preparation

For QuEChERS extraction, 10 g of potato and soil samples or 5 g of potato plant samples was weighed into 50 mL centrifuge tubes. After adding 10 mL of acetonitrile to each tube, the samples were shaken for 5 min. Subsequently, 3 g of anhydrous MgSO_4_ and 2 g of NaCl were added, followed by another 3 min of shaking. The tubes were then centrifuged at 4000 rpm for 5 min, and 1.5 mL of the supernatant was extracted for dispersive solid-phase extraction (d-SPE) purification. For d-SPE, 1.5 mL of supernatant was transferred into a 2 mL centrifuge tube containing specific sorbent mixtures tailored to each matrix: 150 mg MgSO_4_ and 50 mg PSA for potato, 150 mg MgSO_4_ and 50 mg C18 for soil, and 50 mg PSA and 20 mg GCB for potato plant. Each mixture was vortexed for 1 min and centrifuged at 5000 rpm for 5 min. The resulting supernatants were filtered through a 0.22 µm organic filter membrane and transferred into vials for LC-MS/MS analysis.

### 3.8. Instrumental Detection

The study employed Waters Acquity ultra-high-performance liquid chromatography–tandem triple quadrupole mass spectrometry (UPLC-TQD MS/MS) for analysis. Imidacloprid separation was achieved using an Acquity UPLC BEH C18 column, with a mobile phase consisting of acetonitrile (phase A) and water (phase B). The elution gradient used for chromatographic separation is detailed in Supplementary Table S4. Instrumental parameters included a flow rate of 0.3 mL/min, a column oven temperature of 30 °C, and an injection volume of 5 µL. The UPLC-MS/MS method utilized positive electrospray ionization (ESI+) with a 3 kV ion spray voltage and an ion source temperature of 150 °C. Gas parameters for imidacloprid detection included 50 L/h nitrogen as cone gas, 2 × 10^−3^ mbar argon as collision gas, and 600 L/h nitrogen at 350 °C as dry gas. Mass spectrometry conditions optimized for identifying, confirming, and quantifying imidacloprid are detailed in Supplementary Table S5.

### 3.9. Residue Calculation

The imidacloprid residues in the sample were calculated according to the following formula (Equation (1)):

(1) $$R=\frac{C_{cst}\times V_{ist}\times S_{psa}\times V_{fsa}}{V_{isa}\times S_{pst}\times W}$$

Here, R = imidacloprid residue in the sample (mg/kg), C_cst_ = concentration of standard solution (μg/mL), V_ist_ = injection volume of standard solution (μL), V_fsa_ = final volume of sample solution (mL), V_isa_ = injection volume of sample solution (μL), S_psa_ = peak area of the injected sample solution, (μVS), S_pst_ = peak area of the injected standard solution (μVS), and W = weight of the sample (g).

### 3.10. Method Quality Control

The method was validated in accordance with European Commission 2021 guidelines (SANTE, 2021), covering parameters such as specificity, linear range, determination coefficient (R^2^), limits of detection (LOD) and quantification (LOQ), accuracy (% recovery), and repeatability (% RSD). Specificity and selectivity were evaluated using chromatograms of standards, matrix blanks, and fortified samples at the lowest calibrated level [22]. A linear range was established using seven standard solutions (0.005 to 2 mg/kg), with calibration curves generating regression equations and R^2^ values. For accuracy and precision, five replications of three-level fortified samples were analyzed, adhering to SANTE guidelines. Acceptable accuracy and precision ranges were 70–120% recoveries and less than 20% RSD, respectively. The LOD, determined by the lowest detectable level in the calibration curve, and the LOQ, defined as the lowest validated level with adequate recovery and precision, were both established. Matrix effects were assessed by comparing the slopes of calibration curves prepared with pure solvent and matrix standards using the following equation:

ME (%) = (slope matrix/slope solvent − 1) × 100(2)

### 3.11. Statistical Analysis

#### 3.11.1. Calculation of Dissipation and Half-Life of Imidacloprid

The time-dependent pesticide concentration in vegetation or soil is given by the initial pesticide concentration and the pesticide degradation rate in vegetation or soil media and can be described as a first-order dissipation equation (Equation (3)) [22].

(3) $$C_{t}=C_{0}e^{kt}$$

The half-life of imidacloprid was calculated with the following Equation (4) [43].

(4) $$T_{\frac{1}{2}}\;=lnln\;2/k$$

where *C_t_* is the pesticide concentration at time *t* (mg/kg), *C*_0_ is the initial concentration at time zero (mg/kg), and *kt* is the removal rate of the pesticide in the vegetation or soil media.

#### 3.11.2. Dietary Risk Assessment

The study assessed the potential risk of imidacloprid residues in potatoes consumed in China by comparing the national estimated daily intake (NEDI) with the acceptable daily intake (ADI) established by the Joint FAO/WHO Meeting on Pesticide Residues (JMPR) [44]. The NEDI was calculated using the supervised trial median residue (STMR) in potato and soil samples, combined with the average potato consumption data for the Chinese population (Equation (5)). This NEDI was then expressed as a percentage of the ADI to determine the chronic hazard quotient (RQ) (Equation (6)). An RQ below 100% indicates acceptable dietary risk, while an RQ at or above 100% suggests potential concern. The ADI for imidacloprid, as per JMPR guidelines, is 0.06 mg/kg body weight. Detailed calculations for NEDI and RQ, incorporating the average body weight of Chinese residents (63 kg) and their daily potato consumption (69.98 g/person/day), are provided within the study [44].

NEDI = (STMR × Fi)/bw(5)

RQ = NEDI/ADI(6)

#### 3.11.3. Ecological Risk Assessment

The study evaluated the potential ecological risk of imidacloprid residues in the top 10 cm of soil within potato fields in Shandong and Jilin provinces, China. This assessment specifically focused on non-target terrestrial organisms, utilizing toxicity exposure ratios (TER) and risk quotients (RQ). The mean (MSC_mean_) and maximum (MSC_max_) concentrations of imidacloprid in soil were determined. Toxicity data (Table 1) for relevant soil invertebrates, obtained from FAO/WHO and ECOSAR databases, were used to calculate predicted no-effect concentration (PNEC) values based on median lethal concentration (LC50) data [45,46,47]. The ecological risk quotient (RQ) was then calculated using Equation (7) to assess the risk posed by imidacloprid to these soil invertebrates. Risk levels were categorized as low (<0.1), moderate (0.1 ≤ RQ < 1), or high (1 ≤ RQ < 10) based on the RQ values [48].

(7) $${RQ}_{Species(mean\;or\;max)}=\frac{{MSC}_{mean\;or\;max}}{{PNEC}_{spices}}=\frac{{MSC}_{mean\;or\;max}}{{LC}_{50}\times (\frac{1}{AF})}$$

Furthermore, TER values for the soil organisms were computed by comparing the no-observed-effect concentration (NOEC) or PNEC with the MSC_mean_ or MSC_max_ (Equation (8)). Cut-off values established by the European Commission, 5 for chronic and 10 for acute toxicity, were employed to determine the risk threshold for pesticide residues [40,49].

(8) $${TER}_{specie}=\left(\frac{{NOEC}_{Species}\;or\;{PNEC}_{Species}}{{MSC}_{or\;mean}}\right)$$

## 4. Conclusions

A modified QuEChERS method coupled with UPLC-MS/MS was validated for the analysis of imidacloprid residues in potato plants and soil, and revealed high accuracy (recovery: 70.8–95.6%) and excellent linearity (R^2^ ≥ 0.9950). Field studies conducted over two growing seasons in Weifang (Shandong) and Shuangliao (Jilin), China, revealed imidacloprid half-lives of 6.9–26.7 days in plants and 19.8–28.9 days in soil. At harvest, imidacloprid residues in potatoes were below 0.238 mg/kg, and less than 0.149 mg/kg in soil when used at recommended dosages of FS seed treatment formulation. The dietary risk assessment indicated a relatively low risk to human health (39.73%). However, an ecological risk assessment using RQ and TER methods revealed low to moderate risks to earthworms, emphasizing the need for judicious imidacloprid FS use in seed treatment to balance pest control efficacy with environmental safety.

## Figures and Tables

**Table 1:** Validation parameters of imidacloprid in solvent and sample matrices.

Analytes	Matrix	Linear Range (mg/kg)	Regression Equation	R^2^	LOD (mg/kg)	LOQ (mg/kg)	ME (%)
Imidacloprid	Acetonitrile	0.005–2.0	y = 80,331x + 9161.9	0.9996	0.005	0.01	
	Potato	0.005–2.0	y = 23,184x + 3816.5	0.9992	0.005	0.01	−71.1
	Potato plant	0.005–2.0	y = 2890x + 412.97	0.9994	0.005	0.01	−96.4
	Soil	0.005–2.0	y = 22,360x + 3113.9	0.9950	0.005	0.01	−72.2

**Table 2:** Accuracy and precision of imidacloprid in potato, potato plant, and soil matrices.

Imidacloprid	Spiked Level (mg/kg)	Recovery (%)					Average Recovery (%)	RSD (%)
		1	2	3	4	5		
Potato	0.01	80.8	71.0	72.3	74.5	73.0	74.32	5.16
	0.1	89.6	86.1	85.0	87.5	87.0	87.04	1.97
	0.5	89.0	86.8	91.7	89.8	87.7	89.00	2.13
Potato plant	0.01	70.6	73.6	75.4	69.7	64.6	70.78	5.85
	0.1	80.7	72.0	74.6	71.6	80.3	75.84	5.81
	0.5	72.6	70.0	84.0	84.7	85.0	79.26	9.25
Soil	0.01	85.5	119.5	100	75.3	97.8	95.62	17.41
	0.1	87.5	87.2	91.2	85.5	97.5	89.78	5.33
	0.5	73.4	79.8	89.2	100.9	79.2	84.50	12.75

**Table 3:** The terminal residue of imidacloprid in potato.

Time	Place	Potato Variety	Application Dose (g a.i./100 kg Seed)	Mean Residue (mg/kg)
2018	Shandong Province	Virus-free	30	0.059
			45	0.077
2019	Shandong Province	Virus-free	30	0.238
			45	0.778
2018	Jilin province	Holland 7	30	0.029
			45	0.033
2019	Jilin province	Holland 7	30	0.104
			45	0.075

**Table 4:** The terminal residue of imidacloprid in potato field soil.

Time	Place	Potato Variety	Application Dose (g a.i./100 kg seed)	Mean Residue (mg/kg)
2018	Shandong Province	Virus-free	30	0.078
			45	0.118
2019	Shandong Province	Virus-free	30	0.066
			45	0.149
2018	Jilin province	Holland 7	30	0.027
			45	0.021
2019	Jilin province	Holland 7	30	<0.01
			45	<0.01

**Table 5:** The result of the dietary risk assessment for imidacloprid in potatoes.

Food Species	Dietary Intake (kg)	Reference MRL (mg/kg)	Source of MRL	TMDI (mg)	ADI (mg)	RQ (%)
Rice and its products	0.2399	0.05	China	0.011995	ADI × 63	
Wheat flour and its products	0.1385	0.05	China	0.006925		
Other grains	0.0233	0.05	China	0.001165		
Tubers	0.0495	0.239	STMR	0.011831		
Dried beans and bean products	0.016	0.05	China	0.0008		
Vegetables with a light color	0.0915	5	China	0.4575		
Vegetables with a dark color	0.1837	5	China	0.9185		
Pickles	0.0103			0		
Fruits	0.0457	1	China	0.0457		
Nuts	0.0039			0		
Poultry and Meat	0.0795			0		
Milk and milk products	0.0263			0		
Eggs and egg products	0.0236			0		
Fish and shrimp	0.0301			0		
Vegetable oil	0.0327	0.5	China	0.01635		
Animal oil	0.0087			0		
Sugar and starch	0.0044	0.2	China	0.00088		
Salt	0.012	1	China	0.012		
Soy sauce	0.009	2	China	0.018		
Total	1.0286			1.501646	3.78	39.73

**Table 6:** Imidacloprid earthworm toxicity exposure ratio (TER) on Shandong and Jilin potato field soils in China.

		30 g a.i./100 kg Seed		45 g a.i./100 kg Seed	
Province	Species	TER_mean_	TER_max_	TER_mean_	TER_max_
Shandong	*Dendrobaena octaedra*	7.97	7.13	4.29	3.80
	*Allolobophora icterica*	3.92	3.50	2.11	1.87
	*Eisenia foetida*	3.22	2.88	1.73	1.53
Jilin	*Dendrobaena octaedra*	8.91	5.70	36.77	27.14
	*Allolobophora icterica*	4.38	2.80	18.06	13.33
	*Eisenia foetida*	3.59	2.30	14.84	10.95

## Data Availability

The original contributions presented in the study are included in the article/Supplementary Material, further inquiries can be directed to the corresponding author.

## Supplementary Materials

The following supporting information can be downloaded at: https://www.mdpi.com/article/10.3390/molecules29143362/s1, Table S1: Summary of field trial records for imidacloprid dissipation on potato fields; Table S2: Climate and soil characteristics of the experimental potato fields; Table S3: Doses, application times, and sampling schedule for imidacloprid residue tests on potato fields; Table S4: Gradient elution of mobile phase for imidacloprid determination; Table S5: Mass spectrometry conditions for imidacloprid determination.
