# Supplementary material for: Evaluating the Safety of Imidacloprid FS Seed Treatment Use in Potato Production: A Case Study from China
Source: Molecules. 2024 Jul 17;29(14):3362. doi: 10.3390/molecules29143362 (PMC11279475; doi:10.3390/molecules29143362)
Supplement: Supplementary file 1 [file molecules-29-03362-s001.zip › molecules-3098039-supplementary.pdf]

Research Article

Evaluating the Safety of Imidacloprid FS Seed Treatment Use in Potato Production: A Case Study from China

Abdul Kaium [1,2], Chi Wu [1], Yanli Man [1], Xingang Liu [1,*], Fengshou Dong [1] and Youngquan Zheng [1]

[1] State Key Laboratory for Biology of Plant Disease and Insect Pests, Institute of Plant Protection, Chinese Academy of Agricultural Sciences, No. 2, West Yuan-Ming-Yuan Road, Beijing 100193, China; kaium.agrichemistry@sau.edu.bd (A.K.); wuchi@caas.cn (C.W.); sdstzsmyl@163.com (Y.M.); fsdong@ippcaas.cn (F.D.); yqzheng@ippcaas.cn (Y.Z.)

[2] Department of Agricultural Chemistry, Sher-e-Bangla Agricultural University, Dhaka 1207, Bangladesh

* Correspondence: liuxingang@caas.cn; Tel./Fax: +86-10-62815908

Supplementary Tables:

Table S1. Summary of field trial records for imidacloprid dissipation on potato fields.

Location	Start time	Potato varieties	Plot area (m^2)	Replication (times)
Zhuliu Town, Changle County, Weifang City, Shandong Province	2018.8.18	Virus-free	30	3
	2019.3.10		30	3
Jianshe Village, Namu Township, Shuangliao City, Jilin Province	2018.5.12	Holland 7	30	3
	2019.4.15		30	3

Table S2. Climate and soil characteristics of the experimental potato fields.

Provinces	Climatic zone	Soil type	Soil pH	Organic matter (%)	Sunshine hours (h)	Average temp. (°C)	Average precipitation (mm)
Shandong	Temperate: semi-humid	Fluvo-aquic	6.9	2.0	2300-2700	21	134
Jilin	Temperate: continental-monsoon	Black	6.4	2.3	2259-3016	23	116

Table S3. Dose, application time and sampling schedule for imidacloprid residue test on potato fields.

Dosage	Application times	Test sample	Times of sampling after last application
50 mL/100 kg seed potato	1	Final residue (Potato, soil)	Harvest period
75 mL/100 kg seed potato	1	Final residue (Potato, soil)	Harvest period
75 mL/100 kg seed potato	1	Dissipation dynamics (Potato plant)	After application: 1, 3, 5, 7, 10, 14, 21 days
75 mL/100 kg seed potato	1	Dissipation dynamics (Soil)	Before and after application: 2h, 1, 3, 7, 14, 21, 28, 35days
Control	0	Blank control Potato, potato plants, soil	Potato, potato plants, soil

Table S4. Gradient elution of mobile phase for imidacloprid determination

Time	Flow rate	Mobile solvent elution (A: B, V/V)
0 min	0.3 mL/min	10:90
1.5 min	0.3 mL/min	90:10
2.5 min	0.3 mL/min	90:10
2.6 min	0.3 mL/min	10:90
5.0 min	0.3 mL/min	10:90

Table S5. Mass spectrometry conditions for Imidacloprid determination.

Analyte	Parent Ion	Daughter Ion	Dwell time	Cone voltage (V)	Collision energy (V)
Imidacloprid	255.96	175	0.163	40	30
		209*	0.163	40	32

* Quantitative ion
